# Benign and Malignant Breast Tumor Classification in Ultrasound and Mammography Images via Fusion of Deep Learning and Handcraft Features

**DOI:** 10.3390/e25070991

**Published:** 2023-06-28

**Authors:** Clara Cruz-Ramos, Oscar García-Avila, Jose-Agustin Almaraz-Damian, Volodymyr Ponomaryov, Rogelio Reyes-Reyes, Sergiy Sadovnychiy

**Affiliations:** 1 Escuela Superior de Ingenieria Mecanica y Electrica-Culhuacan, Instituto Politecnico Nacional, Santa Ana Ave. # 1000, Mexico City 04430, Mexico; ccruzra@ipn.mx (C.C.-R.); ogarciaa1903@alumno.ipn.mx (O.G.-A.); jalmarazd1401@alumno.ipn.mx (J.-A.A.-D.); rreyesre@ipn.mx (R.R.-R.); 2 Instituto Mexicano del Petroleo, Lazaro Cardenas Ave. # 152, Mexico City 07730, Mexico; ssadovny@imp.mx

**Keywords:** fusion, feature selection, genetic algorithm, mutual information, ultrasound image, mammography image, breast cancer

## Abstract

Breast cancer is a disease that affects women in different countries around the world. The real cause of breast cancer is particularly challenging to determine, and early detection of the disease is necessary for reducing the death rate, due to the high risks associated with breast cancer. Treatment in the early period can increase the life expectancy and quality of life for women. CAD (Computer Aided Diagnostic) systems can perform the diagnosis of the benign and malignant lesions of breast cancer using technologies and tools based on image processing, helping specialist doctors to obtain a more precise point of view with fewer processes when making their diagnosis by giving a second opinion. This study presents a novel CAD system for automated breast cancer diagnosis. The proposed method consists of different stages. In the preprocessing stage, an image is segmented, and a mask of a lesion is obtained; during the next stage, the extraction of the deep learning features is performed by a CNN—specifically, DenseNet 201. Additionally, handcrafted features (Histogram of Oriented Gradients (HOG)-based, ULBP-based, perimeter area, area, eccentricity, and circularity) are obtained from an image. The designed hybrid system uses CNN architecture for extracting deep learning features, along with traditional methods which perform several handcraft features, following the medical properties of the disease with the purpose of later fusion via proposed statistical criteria. During the fusion stage, where deep learning and handcrafted features are analyzed, the genetic algorithms as well as mutual information selection algorithm, followed by several classifiers (XGBoost, AdaBoost, Multilayer perceptron (MLP)) based on stochastic measures, are applied to choose the most sensible information group among the features. In the experimental validation of two modalities of the CAD design, which performed two types of medical studies—mammography (MG) and ultrasound (US)—the databases mini-DDSM (Digital Database for Screening Mammography) and BUSI (Breast Ultrasound Images Dataset) were used. Novel CAD systems were evaluated and compared with recent state-of-the-art systems, demonstrating better performance in commonly used criteria, obtaining ACC of 97.6%, PRE of 98%, Recall of 98%, F1-Score of 98%, and IBA of 95% for the abovementioned datasets.

## 1. Introduction

Breast cancer is a disease that affects women worldwide; it is the most-diagnosed and represents one of the four cases of dangerous types of cancer. Furthermore, it is the leading cause of death in women. An estimated 2.3 million new cases in 2020 indicate that one in every eight diagnoses that year was breast cancer. In 2020, there were an estimated 684,996 deaths from breast cancer, and, by 2040, the number of cases will increase by almost 50% [1,2].

In cancer diagnosis, there are benign and malignant types of tumors. The benign tumors do not spread throughout the body and usually do not reappear when extracted by surgery. The malignant tumors invade the tissue around the breast, and the cancer cells can spread and invade other body organs, causing development of metastasis, one of several complications that can cause the patient to die. Treatment for breast cancer can be highly effective, preventing the progression and eradication of the disease and giving a 90% or higher probability of survival, mainly when it is detected early. Treatment refers to a combination of surgical removal, radiotherapy, immunotherapy, and chemotherapy [3,4].

This study examines different medical imaging techniques, namely, mammography (MG) and ultrasound (US). Images in the first case are obtained by emitting small amounts of radiation. Then, this radiation is absorbed, depending on the density of the tissues. Finally, an image is obtained, depending on the dose of radiation that passes to the different tissues [5]. In the second case, the US images are obtained by emitting US waves to produce the image, where the acoustic impedance plays an important role, since this term is used to describe the resistance to the passage of US energy through a substance or tissue due to its refractive and absorption properties. Because different tissues have different impedances, those with higher impedances appear brighter, as the wave returns with greater intensity, such as in the case of bones. A US sensor also calculates the return time of the wave, meaning near objects are reflected before distant ones and are, accordingly, placed closer to the screen [6,7].

The American College of Radiology (ACR) established a standardized method for describing the perceptual features of a breast lesion contained in medical imaging, such as MG and Computer Tomography (CT). This system, called BI-RADS (Breast Imaging Reporting and Database System), allows one to determine if a mass is benign or malignant according to its features, such as shape, texture, and size, and indicates the probability of each state. Therefore, the patient continues treatment, depending on the diagnosis obtained [6,8,9,10]. Below, we present a brief description of the BI-RADS system.

BI-RADS 0 is assigned when the image does not provide enough information for diagnosis. Prior studies must be requested, and new images are acquired for analysis. BI-RADS 1 characterizes a normal breast in the MG image, i.e., one which does not present suspicious findings. In the category of BI-RADS 2, there are no signs of cancer, but there may be benign findings. BI-RADS 3 to BI-RADS 5 categories express probability values which are greater than 0% and up to less than or equal to 95% of being a malignant neoplasm. BI-RADS 6 expresses that the presence of cancer is confirmed.

Most of the automated CAD (Computer Aided Diagnostic) systems are based on various machine or deep learning strategies, applying deep or handcraft features to obtain superior performance in different applications, such as segmentation and classification. The performance of a CAD system is characterized via commonly used metrics such as Accuracy (*ACC*), Precision (*PRE*), Sensitivity (*SEN*), Specificity (*SPE*), F1-Score, etc. Below, we present a brief review of recently proposed CAD systems that demonstrated excellent performance in terms of those metrics.

Wei et al. [11] used a database collected from Quanzhou First Hospital in Fujian, China. Their system removed the edges of the images, eliminating artifacts. As the handcraft features, they employed the uniform Local Binary Patterns (uLBP), Histogram-Oriented Gradient (HOG), and Grey Level Co-occurrence Matrix (GLCM) texture features. Lastly, two different SVM classifiers, based on the Bayes theorem, separated these features into two classes. As a result, they obtained the following criteria values in binary classification: ACC of 91.11%, SEN of 94.34%, and SPE of 86.49%.

Zhang et al. [12] first segmented the ROI of MG by removing noise, enhancing the image via logarithmic spatial transform and removing the oblique-pectoral muscle, as well as the background. Next, the coefficients of time-frequency spectrum were obtained via fractional Fourier transform; later, those features were reduced via the PCA technique. At the final stage, the classifiers (SVM and k-nearest neighbors) were employed, resulting in the following performance results (in the SVM case): SEN of 92.22%, SPE of 92.10%, and ACC of 92.16%.

Daoud et al. [13] obtained the ROI image using a delimiting box. Next, the classification of US breast lesions was carried out by employing extraction of deep features, using the VGG-19 model and selecting the handcraft features, such as texture (800 features) and morphological features (18 features). Then, the method performed the combination of handcraft features with the deep features by each convolutional layer of the CNN architecture, obtaining an ACC of 96.1%, SEN of 95.7%, and SPE of 96.3%.

Jabeen et al. [14] performed, in their system, several main steps: data augmentation, as well as processing via pre-trained DarkNet-53 architecture by modifying the output layer and extracting the features contained in the *Global Average Pooling* layer. Afterwards, two optimization algorithms were used to extract the best features: Reformed Differential Evaluation (RDE) and Reformed Gray Wolf (RGW). The classification of the obtained features via the cubic SVM reported a PRE of 99.3%.

Heenaye-Mamode et al. [15] developed a convolutional neural network (CNN) to segment and classify distinct types of breast abnormalities, such as asymmetry, calcifications, masses, and carcinomas. Firstly, the Transfer Learning method was carried out on their dataset using the pre-trained model ResNET-50. Then, they employed an enhanced deep learning model by adjusting the learning rate adaptively under variations in error curves. As a result, the novel model achieved a PRE of 88% in classifying these four types of breast cancer abnormalities (masses, calcifications, carcinomas, and asymmetry) in MG images.

Tsai et al. [16] performed BI-RADS classification by using a database of the E-Da hospital in Taiwan and assigning the labels for each image proposed by physicians. The category was assigned according to the proportion of lesion areas in the location, a 224 × 224 block with 36-pixel pitch. The method was based on the EfficientNET deep architecture. Finally, they carried out the classification, obtaining a PRE of 94.22%, SEN of 95.31%, and SPE of 99.15%.

Muduli et al. [17] proposed a CNN model for automated breast cancer classification from different types of images: MG and US. The model contained five learnable convolutional blocks, each containing four convolutional layers and a fully connected layer as a classifier. The model automatically extracted prominent features from the images with fewer tunable parameters. Exhaustive simulation results on MG datasets (MIAS, DDSM, and INbreast) and US datasets (BUS-1 and BUS-2) confirmed better performance against recent state-of-the-art schemes. In addition, data augmentation permitted reducing overfitting. Their CNN model achieved an ACC of 96.55%, 90.68%, and 91.28% on MIAS, DDSM, and INbreast datasets, respectively. Similarly, ACCs of 100% and 89.73% were achieved on the BUS-1 and BUS-2 datasets, respectively.

In their work, Raza et al. [18] presented a CNN architecture with 24 convolutional blocks consisting of 6 convolutional filters, 9 Inception modules, and 1 fully connected layer. They used the RELU, Leaky-RELU, and RELU-clipped activation functions and Batch Normalization. The designed architecture reached an ACC of 99.35%, PRE of 99.6%, SEN of 99.66%, and an F1-Score of 99.6%.

Alsheikhy et al. [19] presented a study that used the AlexNET CNN architecture, employing different classifiers such as K-Nearest Neighbor (KNN), Naive Bayes with the Gaussian kernel, and Decision Tree (DT). DWT was employed for the images in an attempt to denoise White Gaussian Noise. Furthermore, the PCA technique was used to reduce the high-dimensional obtained data. Three private datasets were evaluated: Kaggle Breast Histopathology Images (BHI), CBIS-DDSM Breast Images, and Breast Cancer Wisconsin (BCW). Its average ACC was over 98.6%, and several metrics were greater than 98.0%.

In the study of Zhang et al.  [20], the authors employed standard eight-layer CNN and improved it by integrating two techniques: Batch Normalization (BN) and Dropout (DO). In the final stage, they used Rank-based Stochastic Pooling (RSP). The BDR–CNN model, a combination of mentioned techniques, was hybridized with a two-layer GCN, resulting in a novel BDR–CNN–GCN model. It was utilized in experiments with 322 MG images from the mini_MIAS dataset, and a 14-way data augmentation method was employed. The performance of the novel framework achieved a SEN of 96.20%, SPE of 96.00%, and ACC of 96.10%.

Nagwan et al., in their study [21], generated input images with a pseudocolor technique— Contrast Limited Adaptive Histogram Equalization (CLAHE)—and pixel-wise intensity adjustment. The generated image was composed by the original image in the first channel, and the second channel represented the CLAHE enhanced image. Finally, the last channel contained the obtained pseudocolor image. These images were fed as a backbone of CNN to generate high-level deep features. Next, a processing technique based on Logistic Regression (LR) and analysis (PCA) was applied. The system was evaluated on two datasets: INbreast and mini-MIAS. The proposed CAD system could achieve the highest performance ACC of 98.60% and 98.80% for the INbreast and mini-MIAS datasets, respectively.

The major drawback in the previous studies, where the deep features extraction strategy was employed, is the absence of a procedure for characterizing and selecting the deepest features, which could measure the information significance of these features focusing on the classification performance. The current study proposes a novel fusion strategy for identifying informative features and eliminating irrelevant ones that might degrade the classification performance. Additionally, we have investigated and justified better performance of the designed method in combining the deep features with handcraft features that can guarantee higher classification performance.

To overcome the above issues, we propose an efficient deep learning–handcraft model that is suitable for MG and US breast images. The major contributions of this work are as follows:

- Deep learning and handcraft features are fused via analysis of the lesions’ features in accordance with statistical criteria, guaranteeing a better performance in the diagnosis;
- Two types of studies, of different natures, are used. Use of MG and US images in developed systems justifies the claim of better performance of the novel systems against recent state-of-the-art systems in MG and US databases, whether standalone or combined;
- Several feature fusion algorithms, such as genetic algorithms and mutual information, are employed; they are based on probabilistic methods and appear to demonstrate superior performance in classifying lesions in MG as well as in US images.

The rest of the manuscript is organized in the following sections: Section 2 describes the proposed system and fusion procedure of the proposed features. Section 3 explains the experimental setup to test and performance evaluation results. A discussion of the evaluation is presented in Section 4. Finally, the conclusions of the study are stated in Section 5.

## 2. Materials and Methods

In this study, we used two datasets, one with US images and the other with MG images, as explained below.

### 2.1. Databases Used

**Figure 1:**
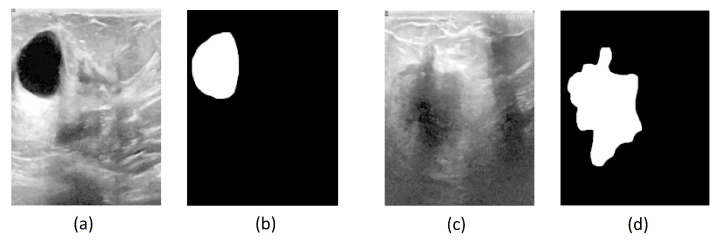
(**a**) Original US image of a benign lesion, (**b**) Benign lesion mask image found in BUSI dataset, (**c**) Original US image of a malignant lesion, (**d**) Malignant lesion mask image found in BUSI dataset.

**Figure 2:**
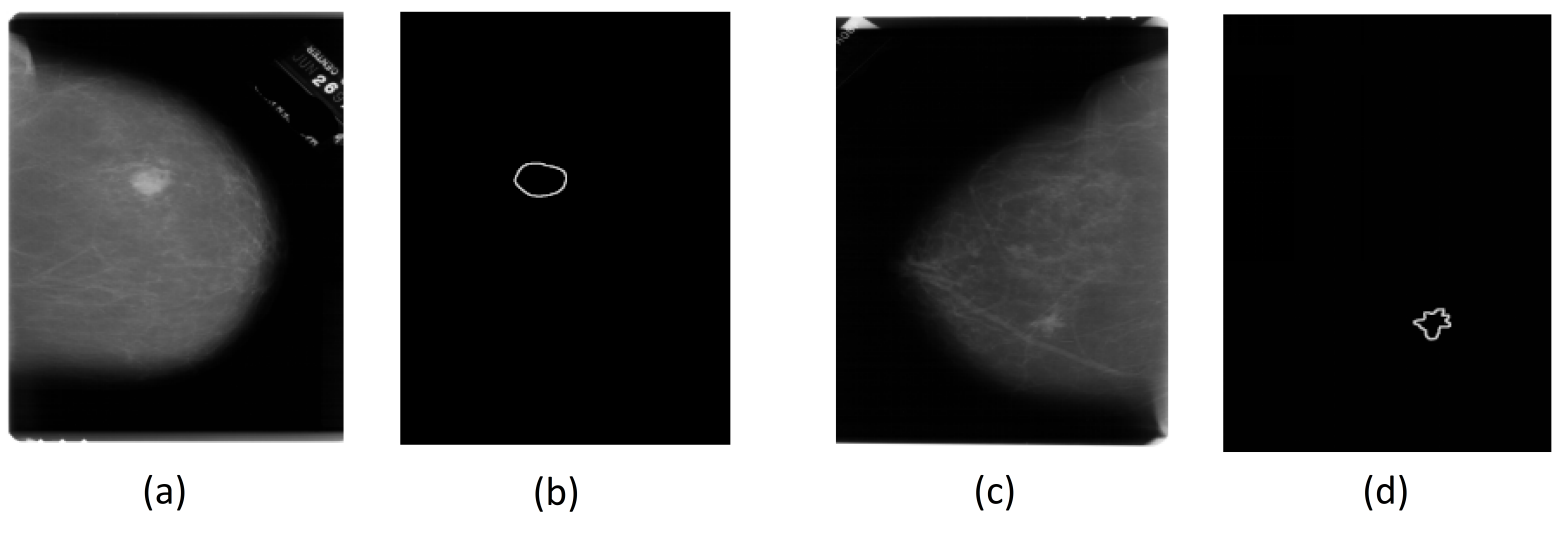
(**a**) Original MG image of a benign lesion, (**b**) Benign lesion mask image found in mini-DDSM dataset, dilated with a filter (7×7) for better visualization, (**c**) Original MG image of a malignant lesion, (**d**) Malignant lesion mask image found in mini-DDSM dataset, dilated with a filter (7×7) for better visualization.

The Breast Ultrasound Images Dataset (Dataset BUSI) is a dataset that was collected in 2018. The collected data consist of 780 breast US images in PNG format, with size 500×500 pixels, including the segmentation masks belonging to 600 patients. Additionally, the images are labeled with one of three classes: normal, benign, and malignant. The database can be obtained online [22]. The mini-DDSM is a current version of the DDSM (Digital Database for Screening Mammography) and is presented in 8-bit JPEG and 16-bit PNG formats. The data are divided into normal, benign, and malignant classes. In addition, the images contain the location of a lesion. The database can be obtained from the following studies and webpage [22,23,24]. Figure 1 and Figure 2 present several examples of benign and malignant lesions from these datasets.

For the employed databases mentioned above, only benign and malignant classes were used to train the proposed CAD system in order to aid the radiologists with the binary classification of breast lesions. Determining if a mammogram is normal or if there are any signs of abnormality is a simple task for a radiologist; however, the classification of a lesion as benign or malignant remains a challenge, even for expert radiologists [25]. Additionally, the proposed system classifies only two categories according to medical classification system BI-RADS-1, in which, if the breast does not contain any lesion, the course to take is an obligatory six-month renewal study. Table 1 shows the distribution of the images according to the classes to which they belonged.

### 2.2. Proposed Method

**Figure 3:**
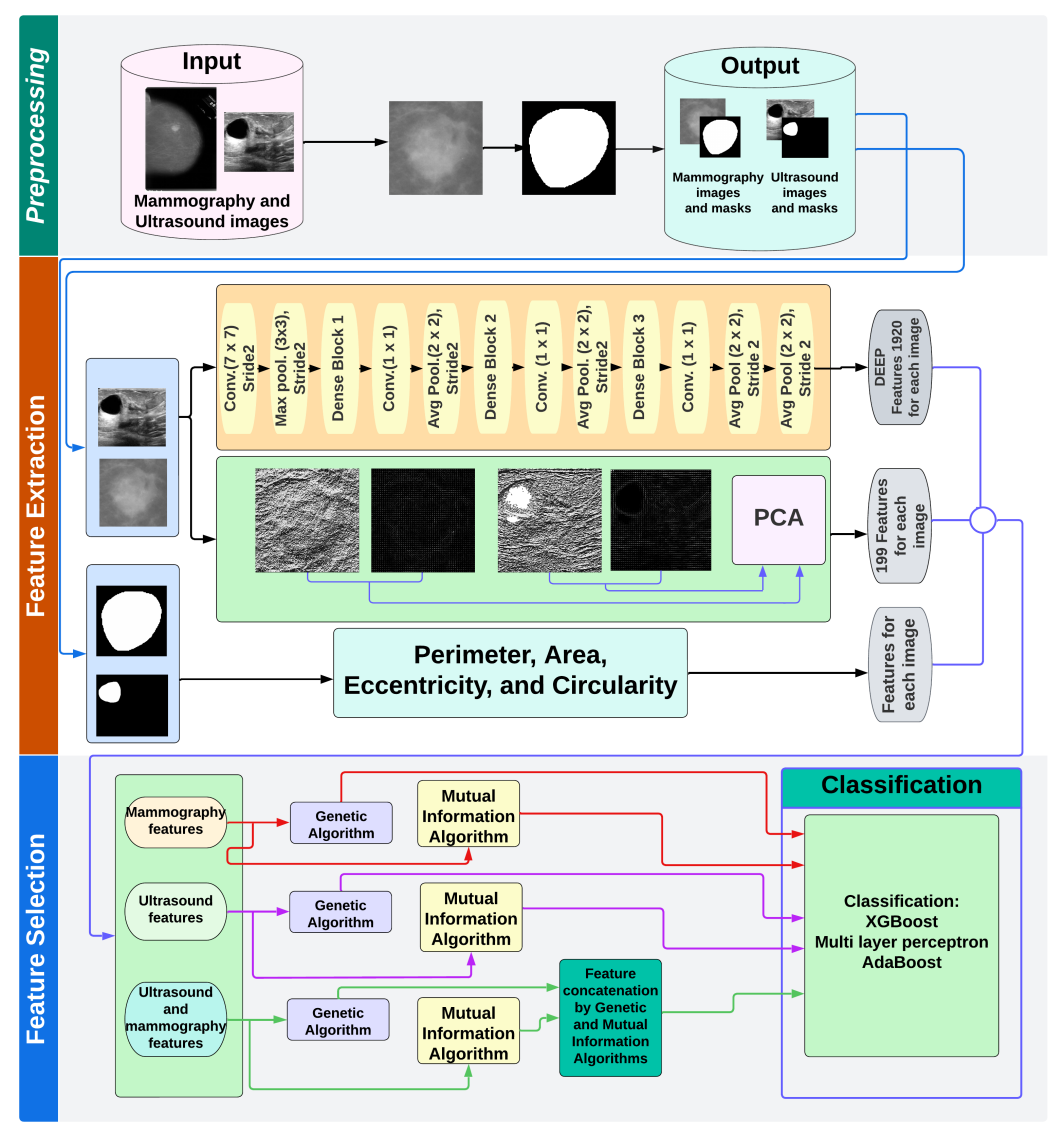
Block diagram of the proposed Computer Aided Diagnostic system.

The designed system presented in Figure 3, called *Deep Breast Fusion System Genetic Mutual Information* (*DBFS_GMI*) CAD system, contains four principal stages: preprocessing, feature extraction, feature fusion, and classification. During the first stage, the segmentation of the MG image is performed, and the US image is manually segmented. Deep and handcraft features are extracted at the system’s second stage. In the case of handcraft features based on the BI-RADS medical system, the shape and texture features, such as area, perimeter, eccentricity, and circularity features, are extracted, as well as HOG and ULBP features. At the same time, deep features are obtained using DenseNET-201 architecture. Then, the deep and handcraft features are concatenated. The third (critical) stage is feature fusion, where all features for two modality images, MG and US, are investigated via genetic algorithm, and mutual information selection based on probabilistic methods is performed, permitting selection of features to produce a better influence on final performance. In the final stage, the classification is performed by employing several classifiers: XGBoost, Multilayer Perceptron, and AdaBoost. In the remaining portion of this section, we explain in detail the operation that is performed in the proposed system.

#### 2.2.1. Segmentation

In the first step of the system, the segmentation procedure is performed by a manual segmentation of the two types of medical images.

The segmentation is carried out by splitting an image into subgroups, i.e., defining the objects that characterize an image. This procedure helps to obtain the Region of Interest (ROI) that should be analyzed, removing several artifacts that do not provide information, such as labels or other elements found in the image [26,27]. Thus, the feature extractor stage does not process the complete input image, reducing the inference time and improving the system’s performance. Image segmentation is a crucial procedure in many deep learning schemes, and our classification system is no exception.

Obtaining the ROI image from US is more difficult due to the noise (*speckle*) contained and low contrast values, due to the nature of the acquisition process [11]. Moreover, the lesion is visible to the specialist due to their trained eye, so a radiologist manually cropped each image and labeled the ROI.

**Figure 4:**
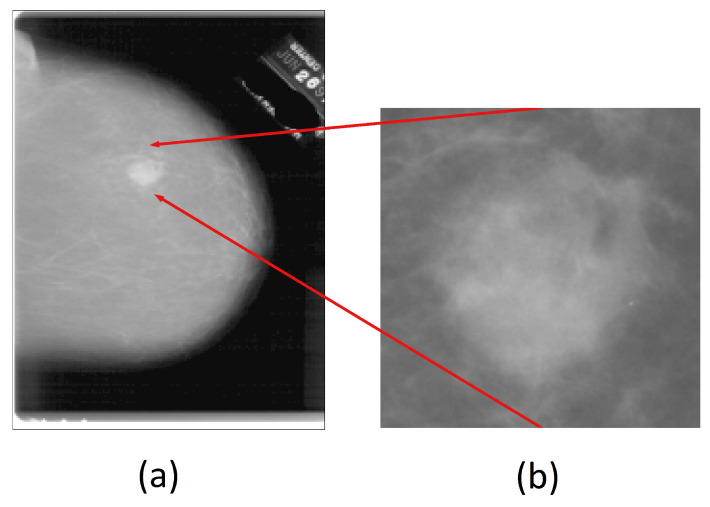
(**a**) Original MG image obtained from mini_DDSM. (**b**) Region of interest (ROI) of a lesion in an MG image.

A specialist manually cropped and labeled the lesion for an MG image (Figure 4). Then, we employed the Suzuki–Abe algorithm to find the contours, in order to delimit the lesion. Next, the bounding box algorithm was used. This method creates a surrounding rectangle to enclose an object, giving us the *x*-axis and *y*-axis coordinates of the limits of the lesion. Then, the given coordinates were used to crop the original images. Finally, we added a 25-pixel tolerance in all four sizes of the generated rectangle, and these coordinates were used to crop the original images. A result of this procedure is shown in Figure 4, where the obtained mass for a lesion contained in an MG image is presented.

An MG image generally varies in contrast, resulting in weak identification of the lesions. As such, for better identification performance, it is necessary to enhance the MG image and highlight its perceptual elements. In addition, contrast is used to make it easier to analyze a lesion. Histogram Equalization was employed in this study to obtain a better contrast in the images. This makes the brightest regions even brighter and the dark ones darker. The procedure was to modify the distribution of the pixels from the original histogram (representative graph of the intensity distribution of the pixels) to a broader distribution range from 0 to 255, where the cumulative distribution is defined as follows:

(1) $$H\left(x\right)=\sum_{y=0}^{x}h\left(y\right),$$

and a new pixel value is reassigned for the further distribution of the equalized image:

(2) $$I_{equalized}\left(x,y\right)=H^{\prime }\left(I\left(x,y\right)\right).$$

Next, we blurred the equalized image using a median filter with a window of (3×3). The purpose of employing a median filter was to eliminate artifacts contained in the image, which have varied intensities [28].

The threshold procedure consisted of employing a mask that delimited the region to be analyzed, thus producing a binary image where the pixels with a higher intensity value than the established threshold have a value of 1, and those that do not meet this criterion take the value zero [29]. This technique was applied to the filtered image from the previous step to obtain a binary image of the ROI:

(3) $$I_{Th}\left(x,y\right)=\left\{\begin{array}{cc} 1\;if\;Img(x,y)>thr; & \\ 0\;\;otherwise. \end{array}\right.$$

**Figure 5:**
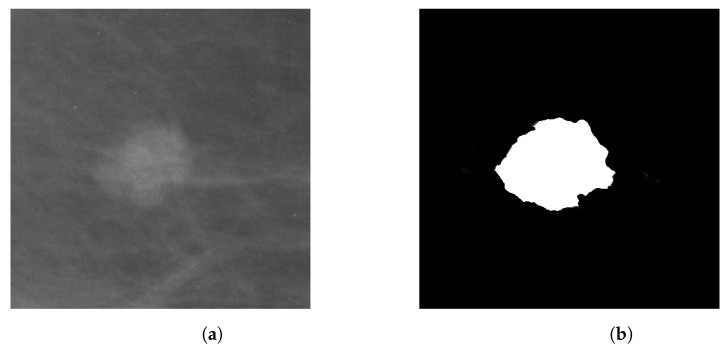
(**a**) ROI of a lesion obtained from MG image. (**b**) Mask of the lesion segmented from an ROI image.

After obtaining the binarized image, we used a median filter of size (9×9) and employed the Suzuki–Abe algorithm to find and delimit the lesion contours. Finally, we labeled the area of the contained objects, choosing the lesion’s most extensive area (see Figure 5).

#### 2.2.2. Feature Extractor Based on Transfer Learning

In this study, the deep learning features were obtained using Transfer Learning via DenseNET-201 architecture, where the ROI images from the dataset US and MG were employed.

Transfer learning is a machine learning technique where a model is used to solve a different task than the one for which it was designed [30,31,32]. This technique is commonly used, since it needs fewer data, as compared to training a CNN from scratch. By applying this technique, deep learning models can be developed to be accurate with shorter processing times. Two main approaches were considered from a pre-trained model: Fine Tuning and Feature Extractor. Fine Tuning implies retraining the model of a specific convolutional layer or convolutional block and substituting the classifier in a particular task, so this method adjusts the model to the current information provided.

On the other hand, the Feature Extractor approach removes the classification block and keeps the extracted features up to the last convolutional layer of the model. These generic features are based on knowledge of the model in a similar task. Finally, these features are analyzed by a classifier.

In the proposed system, we employed the Transfer Learning method on pre-trained architecture, where, in this case, DenseNET architecture trained on ImageNET classification tasks was used, with the assumption that it was employed to classify the specific images over generic features, and, therefore, the architecture should be able to perform as a generic feature extractor.

**Figure 6:**
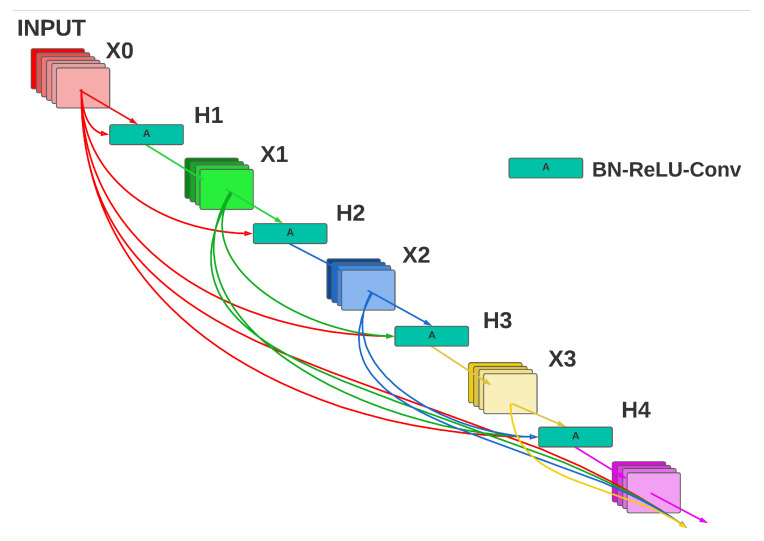
DenseNET architecture.

DenseNET [33] is used commonly, due to its convolutional layers that are densely connected. This feature is advantageous compared to other CNN architectures due to the presence of the vanishing gradient problem, which consists of information being lost or vanishing before reaching the next convolutional block, or even the next convolutional layer. By improving the information dataflow, the dense connectivity of this architecture offers a robust scheme that receives collective knowledge from all previous layers, meaning that the obtained feature maps are shared throughout the architecture. Another benefit is direct access to the gradient values from the loss function and the original image. From the aforementioned highlights of the DenseNET architecture, several authors [34,35,36] have demonstrated that the DenseNET scheme has excellent performance in object recognition applied to different datasets, such as the ImageNET challenge and CIFAR-100 datasets. In Figure 6, a conceptual approach of the DenseNET architecture is presented.

The collective knowledge presented in DenseNET can be expressed as follows:

(4) $$X_{0}\cdots X_{l-1},$$

where $X_{0}$ is the first feature map output, which can be projected in the *l*th layer by all the contained feature maps.

There are feature maps in 0,⋯,l−1 layers that can be composed as a function $H_{l}\left(\cdot \right)$, where three operations are carried out: Batch Normalization, a RELU(·) activation function, and a 3×3 convolution. The sizes of the feature maps are the same within the dense block, since a subsampling is carried out by means of transition layers so that they can be easily concatenated. In the architecture for a dense block with *L* layers, the total number of direct connections between layers is L(L+1)/2. The growth rate *k* is designed to control the number of newly produced feature maps in each layer, because of the concatenation between feature maps. As a result, the total number of feature maps in the layer of a dense block is $k_{0}+\left(l-1\right)*k$, where $k_{0}$ is the number of channels in the input layer.

#### 2.2.3. LBP and HOG Features

The novel system uses handcraft features for texture via Local Binary Patterns (LBP) and, for similar forms, by applying the HOG technique.

Local binary patterns is a descriptor which is widely used to characterize the textures of the images to be analyzed. This is achieved by scanning the pixels through a sliding window and generating a binary code by considering the intensity of the central pixel and comparing it with the intensity of its surrounding neighbors. The result is equal to one if the value is greater; otherwise, it results in zero. To carry out LBP, the radius *r*, which is the distance between the central pixel and the neighbors to be evaluated, and the number of points *p* in a circularly symmetric neighborhood to be considered are used as parameters [37,38,39]. The representation of the LBP operator is defined as follows:

(5) $$LBP_{p,r}=\sum_{i=0}^{p-1}s\left(I\left(x_{i},y_{i}\right)-I\left(x_{c},y_{c}\right)\right)2^{i},$$

where the s(·) function is denoted by:

(6) $$s\left(x\right)=\left\{\begin{array}{cc} 1\;if\;x<0; & \\ 0\;\;otherwise. \end{array}\right.$$

Additionally, $x_{c}$ and $y_{c}$ are the coordinates of the central pixel, and $x_{i},y_{i}$ are the coordinates of its *i*th neighbor within input image *I*.

**Figure 7:**
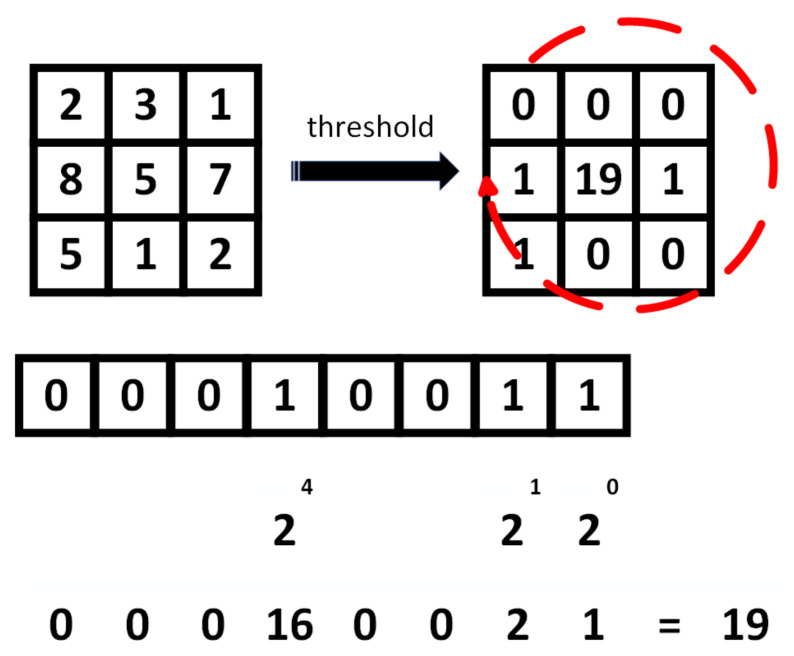
Process of generation by LBP for the central pixel.

To obtain local binary patterns, the following is carried out for each step of the sliding window, which compares the value of the central pixel with each of the neighbors. We start with the pixel at the top right and move clockwise. The comparison is made by taking the intensity of the central value and then subtracting the intensity of the neighboring pixel. If the difference is 0 or less than zero, that is, a negative number, its values are close to zero. On the contrary, if the value of the subtraction is greater than zero, the value is 1. These values are stored as a binary array, and converting them to decimal gives us the new value of the central pixel. Figure 7 illustrates how the value for the central pixel is generated in the LBP method.

Using this new matrix, the histogram is formed, where the values range from 0 to 255, having 256 bins. A histogram *H* of length *K*, calculated from an image *I* of width *M* and height *N*, is defined by the following:

(7) $$H\left(K\right)=\sum_{x=1}^{M}\sum_{y=1}^{N}LBP_{\left(p,r\right)}\left(I(x,y)\right)=k,$$

where K∈[0,1,⋯,k−1] and is the *k*th bin of the histogram.

**Figure 8:**
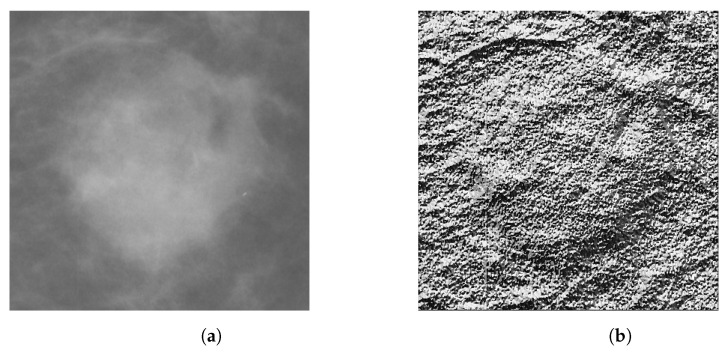
(**a**) ROI image obtained from MG image; (**b**) Generated LBP image of (**a**), where one can see the texture of the lesion.

Figure 8 presents a generated LBP image, obtained from the ROI of an MG image.

HOG is known as a shape descriptor. Using the magnitude and angle of the gradient, the features are calculated [40,41,42].

The gradient is calculated in the vertical and horizontal direction of the pixel (x,y), that is, given by the following:

(8) $$G_{x}\left(x,y\right)=I\left(x+1,y\right)-I\left(x-1,y\right);$$

(9) $$G_{y}\left(x,y\right)=I\left(x,y+1\right)-I\left(x,y-1\right).$$

The magnitude is calculated by the following:

(10) $$G\left(x,y\right)=\sqrt{G_{x}{\left(x,y\right)}^{2}+G_{y}{\left(x,y\right)}^{2}},$$

and the direction is as follows:

(11) $$\alpha \left(x,y\right)=tan^{-1}\left[\frac{G_{y}\left(x,y\right)}{G_{x}\left(x,y\right)}\right].$$

After obtaining the magnitude and angle gradient matrices, they are divided into 8×8 blocks, and the histogram is calculated for each block with 9 containers, where each container has an angle range of $20^{\circ }$ and the value of the direction of the gradient is assigned to its pixel. In this way, we count the direction of the pixel gradient that is in the range of each cell of the block:

(12) $${\#}_{bins}=9\;from\;\left[0^{\circ },180^{\circ }\right].$$

In Equation (12), the bin and its value that is provided to each bin are presented. The bins are numbered 0 through B−1 and have width w=180/B. $bin_{i}$ has boundaries (wi,w(i+1)) and center $c_{i}=w\left(i+1/2\right)$. A pixel is characterized by magnitude μ and orientation Θ. The bin and the value that is provided to each bin are given by the following equations:

(13) $$j=\left[\frac{\Theta }{w}-\frac{1}{2}\right];$$

(14) $$V_{j}=\mu \left[\frac{\Theta }{w}-\frac{1}{2}\right];$$

(15) $$V_{j+i}=\mu \left[\frac{\Theta -C_{j}}{w}\right].$$

Once obtaining all the histograms of 9 bins for each cell, 4 cells (2×2) overlap the cells, with a stride of 8 pixels together, forming a block for a feature vector from the 36:

(16) $$fb_{i}=\left[b_{1},b_{2},b_{3},\cdots ,b_{i}\right].$$

**Figure 9:**
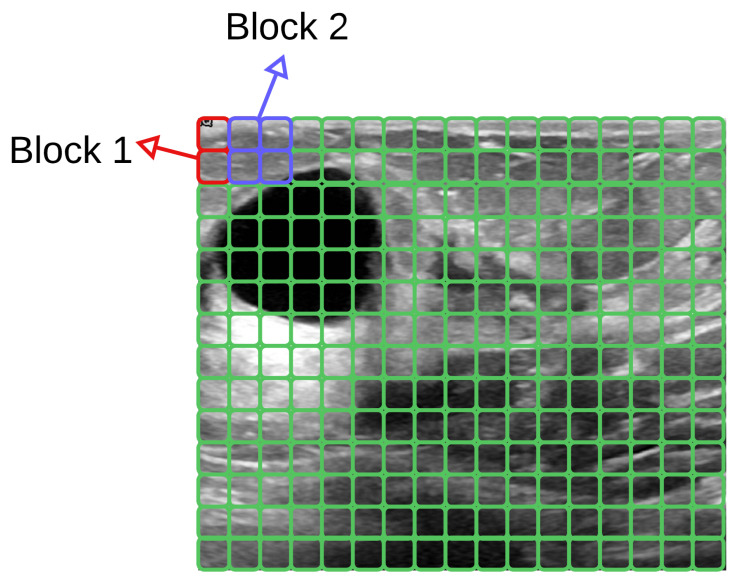
Explanations of how 4 cells (in 2×2) overlap the cells with a stride of 8 pixels together, forming a block.

Figure 9 explains how 4 cells (in 2 × 2) overlap the cells with a stride of 8 pixels, together forming a block.

Then, the values are normalized as follows:

(17) $$fb_{i}\leftarrow \frac{fb_{i}}{\sqrt{∥fb_{i}^{2}∥+\epsilon }},$$

where ϵ is a value up to $1\times 10^{-5}$ that is added to the square of fb to avoid division by zero.

To normalize, the value *k* is first calculated by the following formula:

(18) $$k=\sqrt{b_{1}^{2}+b_{2}^{2}+b_{3}^{2}+\cdots +b_{i}^{2}}.$$

Then, the normalization is obtained using the following equation:

(19) $$fb_{i}=\left[\frac{b_{1}}{k},\frac{b_{2}}{k},\frac{b_{3}}{k},\cdots ,\frac{b_{i}}{k}\right].$$

This normalization is performed to reduce the effects of changes in contrast between images of the same object.

**Figure 10:**
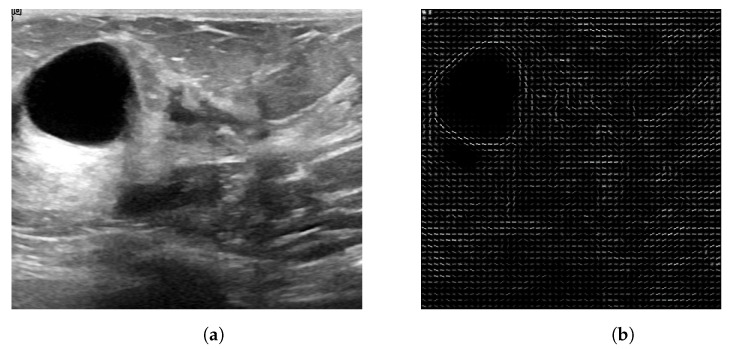
(**a**) ROI image obtained from US image; (**b**) Generated HOG image of (**a**), where one can see the texture of the lesion.

After calculating the LBP texture features and HOG shape features, a feature vector for each descriptor is obtained. The HOG features describe shape patterns, employing the gradients and their directions to help, for both the US and MG images, in the characterization of the edges (*shapes*) of a lesion. LBP features characterize the texture of a lesion, since it is different from breast tissue. Therefore, by employing LBP features, we can expose the texture spotlights of the lesions and the pixels that compose them. Figure 10 presents the generated HOG image, where one can see the texture obtained for the lesion.

#### 2.2.4. Principal Component Analysis

According to the block diagram of the designed system (Figure 3), if the groups of features obtained by descriptors HOG and LBP are redundant, then they could affect the classification process. We proposed to use the Principal Component Analysis (PCA) procedure that can finally reduce a concatenated vector to 199 new components. Below, we explain the PCA technique and its implementation.

PCA is an unsupervised dimensionality reduction technique that converts a dataset into a new smaller dataset, called orthogonal components (*vectors*), preserving the fundamental properties of the original dataset. The goal of PCA is to find the space that represents the direction of maximum variance in the given data [43,44].

To apply the PCA technique to a dataset, the following operations should be performed. First, the average is calculated:

(20) $$\mu \left(x\right)=\sum_{n=1}^{N}\frac{x_{n}}{n}.$$

Then, the variance and the covariance are calculated:

(21) $$\sigma^{2}\left(x\right)=\frac{\sum \left[{\left(x_{i}-\mu \right)}^{2}\right]}{n-1};$$

(22) $$COV\left(X,Y\right)=\frac{\sum \left(x-\mu_{x}\right)\left(y-\mu_{y}\right)}{n}.$$

With these data, the covariance matrix can be determined. The covariance matrix is a square matrix where the variances of the variables are on the diagonal, and the non-diagonal elements consist of the covariances that exist between all pairs of variables possible. This matrix is symmetric.

If the covariance value in the matrix has a positive value, this means that a positive correlation exists between the two variables, while a negative value indicates a negative correlation; if the value is equal to zero, they are not correlated and are statistically independent (for a normal distribution).

This is equivalent to the following:

(23) $$COV\left(X,Y\right)=E{\left[(X-E[X])(Y-E[Y])\right]}^{T}.$$

Using the covariance matrix, eigenvectors (*direction*) and eigenvalues (*magnitude*) can be found. To find the eigenvalues and eigenvectors of a matrix, let us perform the following steps.

The characteristic equation of the matrix is calculated by solving the following determinant:

(24) det(A−λI).

The roots of the characteristic polynomial obtained in the previous step are found. These roots represent the eigenvalues of the matrix:

(25) det(A−λI)=0→λ.

The eigenvector of each eigenvalue is calculated. To do this, the following system of equations should be solved for each eigenvalue:

(26) A−λI=0.

The dimensionality reduction is then obtained by keeping only those axes (*dimensions*) that represent most of the variance, discarding all the others. The PCA space consists of k principal components, where the first principal component of the PCA space represents the direction of the maximum variance of the data, the second principal component has the second largest variance, and so on.

**Figure 11:**
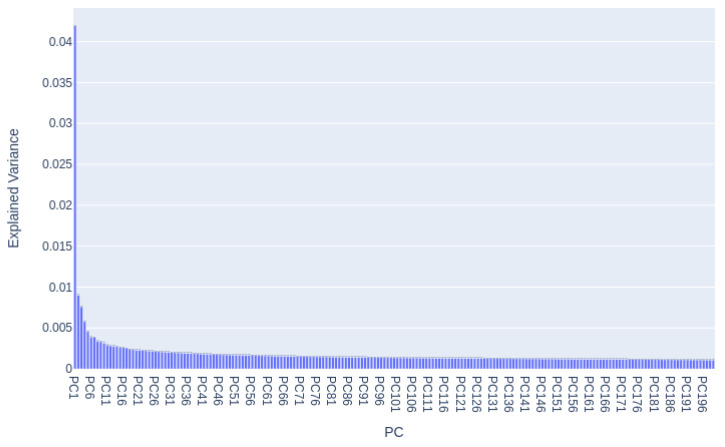
Dependence of variances for PCA components in the selection of HOG and ULBP features.

As the feature vectors of the HOG and LBP descriptors have redundant components, the PCA technique was applied to them after concatenating these two vectors, finally obtaining 199 components (*new features*). The PCA technique represents the features with a minimum number of dimensions without losing their value properties, resulting in the most important information that can be used in the classification stage. Figure 11 explains the number of components chosen that contain the most information of the data. These components are able to represent the HOG and ULBP features.

In the proposed system, different handcraft features are used. We explain these features below.

#### 2.2.5. Shape Features

In the proposed method, we used the features that BI-RADS considers for determining if a lesion is malignant or benign by describing perceptual features.

**Figure 12:**
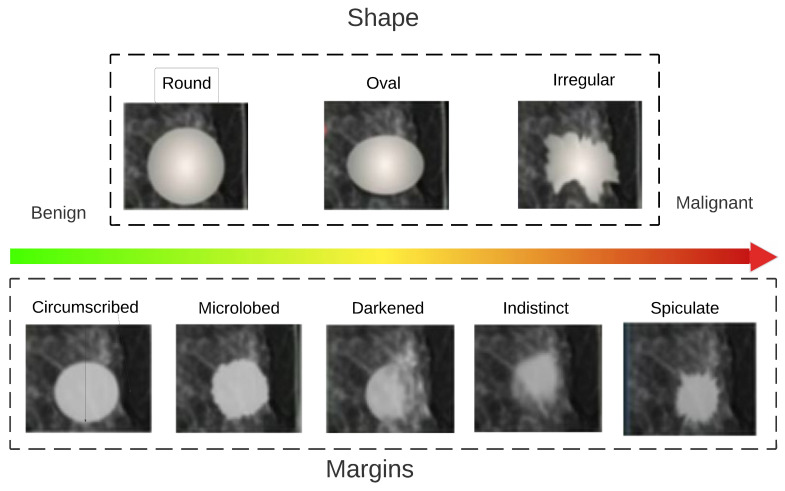
Perceptual description of a mass based on the BI-RADS medical classification algorithm.

The sample classification in Figure 12 shows that a benign mass is round, it is represented by the circumscribed margin, and its density is fatty. In contrast, a malignant lesion has an irregular shape, it is speculated, and its density is high. These features are important, since this study employed them to obtain handcraft-type characteristics and support what a specialist observes during the process of the diagnosis. These features are described as follows:

*Area*—The totality of pixels in a binary ROI that correspond to the binarized region of the lesion, returned as a scalar:

(27) $$Area=\sum_{x=1}^{m}\sum_{y=1}^{n}I_{bin}\left(x,y\right);$$

*Perimeter*—The number of pixels around the region of the lesion:

(28) $$Perimeter=\sum_{i=1}^{m}\sqrt[{2}]{\left(x_{1}-x_{i-1}\right)+\left(y_{1}-y_{i-1}\right)};$$

*Eccentricity*—The relationship that exists between two axes within the lesion, with the ones with the longest and shortest lengths calculated using central moments:

(29) $$Eccentricity=\frac{{\left(\mu_{0,2}-\mu_{2,0}\right)}^{2}+4\mu_{1,1}}{A};$$

*Circularity*—The roundness of the lesion, returned as a structure with a circularity field. The structure contains the circularity value of each object of the input image. The circularity value is calculated as follows [45]:

(30) $$Circularity=\frac{4\pi \cdot Area}{Perimeter^{2}}\;.$$

### 2.3. Feature Selection

According to the scheme of the designed system presented in Figure 3, after obtaining the vector of 2123 hybrid features (deep learning, HOG, LBP, etc.), the selection procedure is performed. In this study, two statistical methods were employed, i.e., techniques based on the genetic algorithm and the mutual information selection algorithm.

#### 2.3.1. Genetic Algorithm

Feature selection is essential to decrease computational complexity by reducing the number of features to be processed. Additionally, this operation can improve system performance by selecting the best features and eliminating features that can cause misclassification. Feature selection employing a genetic algorithm is based on an evolution that consists of finding the feature that best adapts to the environment, as characterized by performance of a chosen classifier [46,47,48,49].

The selection of the features by the genetic algorithm is performed as follows:

1. The initial individuals are produced;
2. A score is determined for the individuals in the population for the predictive model;
3. Selection of the genetic material of best-adapted features passes as a vector. Crossover is applied, where individuals give a part of the chromosomes to create a new individual, and mutations are applied by randomly switching some features on and off;
4. The algorithm runs for a set number of generations (*iterations*). Finally, the result is the group of selected features that are optimal members of the population, according to performance of the selected classifier.

**Figure 13:**
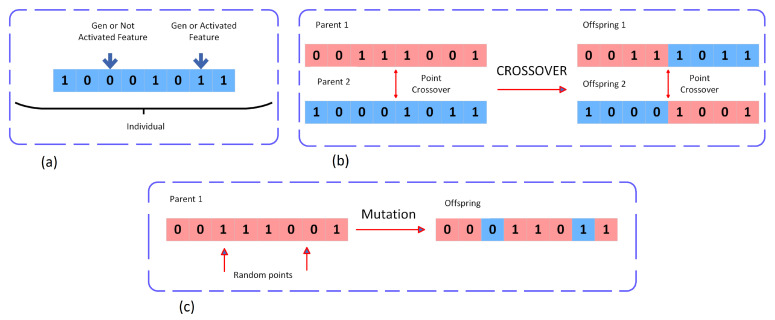
(**a**) Representation of an individual (*gene*); (**b**) Illustration of the crossover operation; (**c**) Mutation operation.

In the designed system, the selection of features was performed as follows:

1. Start by creating a random population of 500 individuals, where the individuals represent subsets of features through a binary string. Each binary digit (*gene*) represents the presence (1) or absence (0) of a given feature in the described way (see Figure 13a);
2. The chosen classifier (*decision tree*) works as the evaluator of the individuals, estimating accuracy of the selected features. For cross-validation, the *5-fold* algorithm his used for the evaluation. The individuals that have obtained the best performance move to the next iteration;
3. The individuals with the best performance chosen in the previous step are mutated and crossed, and those with the lowest performance are eliminated.

The crossing operation uses two chosen individuals with satisfactory performance in the evaluation. They exchange genes in proportion to a determined percentage. In this study, it was equal to 50%, which was designated as a crossover point. The crossing operation is explained in Figure 13b.

The mutation is carried out by randomly changing the individual’s chromosomes. Figure 13c explains the mutation operation.

After these two operations, the new individuals that make up a new population are evaluated by the estimator by using the fitness (*accuracy*) criterion. This process is repeated for every iteration, starting from the evaluation, improving the fitness of the individuals until an iteration where the same accuracy value is maintained, which is the stopping condition of the process (see Table 2). The result of this process is the found binary vector, which indicates the best features with a value of 1.

The *evolutionary algorithm* in this study was as follows:

(31) (μ+λ),

where μ is the number of individuals to select for the next generation and λ is the number of children to produce in each generation. The algorithm was evaluated 35 times. We saw that some features were changed, but not all, in each evaluation. One can see (Table 2) that the accuracy obtained by the classifier did not change significantly in all cases.

#### 2.3.2. Mutual Information

Mutual information is based on Shannon entropy, which measures the dependence or mutual information between two random variables (X,Y) [50,51,52]. It measures the amount of information obtained about one random variable by observing another variable; in other words, it determines how much we can know about one variable by taking into account another.

In machine learning, mutual information measures to what extent information (i.e., presence or absence thereof) of a feature contributes to making the correct prediction on *Y*.

The mutual information between two variables is a non-negative value. It is equal to zero if two random variables are independent, and higher values mean greater dependence. The following formula gives the entropy (i.e., Shannon’s) information:

(32) I(X;Y)=H(Y)−H(Y∣X),

where I(X;Y) is the mutual information, H(Y) is the entropy for *Y*, and H(Y∣X) is the conditional entropy for *Y* given *X*. We can calculate the mutual information as follows:

(33) $$I\left(X;Y\right)=\sum_{x\epsilon X,y\epsilon Y}p\left(x,y\right)log\frac{p(x,y)}{p(x)p(y)}\;.$$

As such, there are joint probabilities (the probability of two things occurring at the same time) and there are marginal probabilities (the probability of just one occurring); the two sums ensure that we include all possible combinations of the variables.

This new set of selected features was then evaluated to obtain the MI among all obtained features, guaranteeing the selection of the most informative data. The study in [53] proposed a K-Nearest Neighbor estimator using the Chebyshev distance between all features against one:

(34) $$d_{chebyshev}=\max_{i}\left(\right|x_{i}-y_{i}\left|\right).$$

Therefore, the MI measure is obtained by the following:

(35) $$I\left(X,Y\right)={\langle \log\frac{p(x_{i},b_{i})}{p\left(x_{i}\right)p\left(b_{i}\right)}\rangle }_{i}.$$

After applying Equation (35), a vector containing the value MI of each feature among all the processed features is created. In this work, we only selected the 26 most informative features, discarding the other ones with low MI values.

#### 2.3.3. Random Undersampling

One of the main problems seen in the development of CAD systems is the imbalanced numbers of samples in the datasets that are used in the development of the machine learning model. A problem arises when having a more significant number of samples in a single class. Applying such imbalanced classes in the classification stage, the class with the greater number of data can be favored or biased to be predicted correctly, and, on the other hand, the classifier is not able to predict the minority class well.

In reality, the model needs to classify or learn the patterns correctly. For this, we need a similar amount of data for all classes. There are several techniques to solve this problem. In this study, the random undersampling (*RUS*) procedure was used, which consists of randomly eliminating samples from the majority class to have a similar and balanced number of samples, making the predictions of the model accurate, since no class is being favoured [54].

### 2.4. Classifiers

After obtaining the best features for each type of image and the fusion of both, three different classifiers were used: XGBoost, AdaBoost, and MLP, to determine the classes to which images belonged, resulting in a prediction of either benign or malignant for each lesion.

#### 2.4.1. XGBoost

Extreme Gradient Boosting (XGBoost) is a distributed and scalable Gradient Boosted Decision Tree (GBDT). It reinforces parallel trees and applies to regression problems and classification. XGBoost includes concepts such as supervised machine learning, which uses datasets with features to predict labels. Decision trees are based on forecasting a label by evaluating the if-then-other true/false question tree and estimating the minimum number of questions needed to evaluate the probability of making a correct decision [55,56].

Gradient Boosted Decision Tree (GBDT) ensemble learning algorithms combine several machine learning algorithms to obtain a better model. This concept is formalized as a gradient descent algorithm on an objective function. Gradient boosting sets specific results for the next model, so as to minimize errors. The predicted results are based on the error gradient for the prediction. For a dataset $D=(x_{i},y_{i})$ with *x* examples and *y* features, it uses *K* additive functions to predict the output:

(36) $$\dot{y}=\sum_{t-1}^{k}ft\left(x_{i}\right),$$

where the prediction is the sum of *K* trees to predict the output of each datapoint $ft(x_{i})$. Each new tree corrects the errors of the previous one, leading to the objective given as follows:

(37) $$L^{\left(t\right)}=\sum_{i=1}^{n}l\left(y_{i},\;{\hat{y_{i}}}^{\left(t-1\right)}+ft\left(x_{i}\right)\right)+\Omega \left(ft\right),$$

where *l* is the loss function that measures the difference between the prediction $\hat{y_{i}}$ and the target $y_{i}$, and Ω penalizes the complexity of the model.

(38) $$L^{∼(t)}=\sum_{i=1}^{n}\left[g_{i}f_{t}\left(x_{i}\right)+\frac{1}{2}h_{i}f_{t}^{2}\left(x_{i}\right)\right]+\Omega \left(ft\right).$$

#### 2.4.2. AdaBoost

The algorithms connected with this approach change the weights of the votes and solve many of the practical problems of the first boosting algorithm. Their rationale is based on the usage of several classifiers, in this case, decision trees, and combining them to create a strong classifier [57,58].

After initially setting the weight D(i) and setting D(i)=1/N, where we assign the same weight value to all samples by dividing each one by the number of samples, the samples have equal importance. Then, a T-training is performed. Next, the error rate is calculated using the following equation:

(39) $$\epsilon_{t}=\sum_{i=1}^{n}D_{t}\left(x_{i}\right)h_{t}\left(x_{i}\right)\neq y_{i}.$$

We use the total error to determine how each classifier fits the data of the previous classifier, since it will be adjusted successively; this performs Adaptive Boosting, where the last model attempts to correct the errors of the first one, until the training on the complete dataset is correctly predicted or the maximum number of classifiers is reached:

(40) $$\alpha_{t}=\frac{1}{2}ln\frac{1-\epsilon_{t}}{\epsilon_{t}}.$$

Then, the sample weights are updated:

(41) $$D_{t+1}\left(x_{i}\right)=\frac{D_{t}\left(x_{i}\right)}{Z_{t}}exp\left\{-\alpha_{t}y_{i}h_{t}\left(x_{i}\right)\right\}.$$

After circulating several times, the weak classifiers are obtained, and the strong classifier is formed:

(42) $$H\left(x\right)=sign\left(\sum_{t=1}^{T}\alpha_{t}h_{t}\left(x\right)\right).$$

#### 2.4.3. Multilayer Perceptron

Multilayer perceptron (MLP) attempts to mimic how the human brain processes information in a computer [59]. It contains several layers, which are commonly fixed to three: an input layer, a hidden layer, and an output layer. Thus, the general structure of a perceptron is described as follows. First, it receives *n* features as inputs, where $x=\{x_{1},x_{2},\cdots ,x_{n}\}$ and the n value denote the vector dimensions of the features. Then, those features are related to a weight *w* and are updated using the back-propagation algorithm [60]. The idea is to obtain a value that regulates each feature. Finally, the features are fed to an activation function to produce the perceptron output. This function is described as follows:

(43) $$u\left(x\right)=f\left(\sum_{i=1}^{n}w_{i}x_{i}\right),$$

where f(·) is the activation function, *w* is the weight to be updated, and *x* is the feature evaluated. Since the weights and the output of the activation function are updated, the input data should be separated (i.e., classified in a binary class).

### 2.5. Algorithm Summary

Let us explain all proposed and presented procedures used in the *DBFS_GMI* CAD system in the form of an algorithm for extraction features for MG and US images. The proposed CAD system consists of four principal stages: (a) preprocessing, (b) handcraft and deep learning features extraction, (c) feature fusion, and (d) classification. In the first stage, artifacts are removed, depending on the nature of the medical images; for the US and MG images, the physician should manually segment the ROI. First, enhancement by histogram equalization is performed for the MG images, and then, by using a median filter, the Suzuki–Abe algorithm, and a thresholding method, a binary image is obtained. We then extract the ROI using the boundary box algorithm. In the second stage, shape and texture features are extracted using Equations (1)–(30). The ROI image is, finally, processed by the chosen CNN architecture, whose features are concatenated for the following steps. In the third stage (feature fusion), the MG and US features are selected using genetic and mutual information algorithms. The found compound features (Equations (31)–(35)) are employed in the class separation. Finally, fused features are used to classify benign and malignant lesions using three different classifiers in the classification stage. Algorithm 1 presents the details of the mentioned processes for both sets of images.
**Algorithm 1** Algorithm summary of the *DBFS_GMI* CAD System.**Require:** 
Image (a) Preprocessing1:Input: *I*2:Apply Fill the gaps I(x,y).3:Apply create Bounding Box by the image that circle the lesion I(x,y).4:Crop according to the coordinates of the bounding box Im(x,y)5:Output: RIm(x,y)
6:Input: RIm(x,y)7:Apply Equalizes the histogram of the image, this improves the contrast RIm(x,y)8:Apply median filter, size = 5 × 5 to improve the edges of the lesion RIm(x,y)9:**for** RIm(x,y) **do**10:    **if** RIm(x,y)≥IL11:        Assign 1 to IThL(x,y)12:    **else if** RIm(x,y)≤IL **then**13:        Assign 0 to IThL(x,y) **then**14:    **end if**15:**end for**16:Apply median filter, size = 9×9 to IThL(x,y)17:Apply apply the Suzuki–Abe method to IThL(x,y)18:Apply the label of the Suzuki–Abe method to find the mask of the lesion IThL(x,y)19:Output: IMask
 (b) Feature Extraction20:Input: IMask21:Compute Area, Perimeter, Circularity, Diameter and Eccentricity from Equations (27)–(30)22:Output: *S handcraft features*
23:Input: RIm(x,y)24:Apply HOG technique to RIm(x,y) from Equations (5)–(7)25:Apply LBP technique to RIm(x,y) from Equations (8)–(19)26:Concatenate for $f^{\prime }=\left(x_{1}^{\prime },x_{2}^{\prime },...,x^{\prime }n\right)$ and $g^{\prime }=\left(y^{\prime }1,y_{2}^{\prime },...,x^{\prime }m\right)$ an obtain Fused Texture features27:Apply PCA to HL from Equations (20)–(26)28:Output: *T Fused Texture features*
29:Input: RIm(x,y)30:Load the weights *W* from selected DenseNET-201 architecture31:Apply the weights *W* to RIm(x,y)32:Obtain the *D* deep learning features from (avg_pool)33:Output: *D deep learning features*
34:Input: *S*, *T*, *D*35:Apply S∪T∪D to the extracted features36:Output: *F Full set of extracted features*
 (c) Feature Fusion37:Input: F for Mammography or Ultrasound38:Apply Mutual Information from Equations (32)–(35)39:Output: MF selected features
40:Input: F for Mammography or Ultrasound41:Apply Genetic algorithm42:Output: UF selected features
43:Input: *F* for Mammography and Ultrasound44:Apply Genetic algorithm and Mutual Information45:Apply UF∪MF to the selected features
 (d) Classification46:Input: UF selected features and MF selected features47:Apply UF∪MF to the extracted features48:Apply Class Separation Using the three classifiers49:Output: Diagnostic of the image *I*.

## 3. Results

### 3.1. Experimental Setup

The described method was performed on a PC with AMD EPYC^®^ 7532, 16 GB RAM, NVIDIA GeForce^®^ 3090 with 24 GB RAM, running a Linux 64-bit operating system, Python 3.10, and the libraries Keras 2.9.0 [61], scikit-learn [62], sklearn-genetic-opt, and imbalanced-learn [63].

### 3.2. Metrics

The evaluation phase is crucial, since it is how we observed the performance of our diagnostic model. This study used recognized and commonly used endpoints for breast cancer screening. These evaluation parameters were accuracy, precision, specificity, sensitivity, and F1-Score. These metrics were obtained by means of a confusion matrix, where the parameters provided were True Positive (*TP*), True Negative (*TN*), False Positive (*FP*), and False Negative (FN) [54,64]. True Positive (*TP*): These are the cases in which the actual datapoint is 1 (*True*), and the prediction is also 1 (*True*); the prediction is correct. True Negative (*TN*): These are the cases in which the actual datapoint is 0 (*False*) and the forecast is also 0 (*False*); the prediction is correct. False Positive (*FP*): These are the cases in which the actual datapoint is 0 (*False*), but the prediction shows that it is 1 (*True*); that is, the prediction is wrong. False Negative (*FN*): These are the cases in which the actual datapoint indicates that it is 1 (*True*), but the forecast is 0 (*False*); the prediction is incorrect. The accuracy value measures the total number of correct predictions on all the elements evaluated; that is, it tells us the percentage that the system evaluated correctly:

(44) $$Accuracy=\frac{tp+tn}{tp+tn+fp+fn}.$$

Sensibility, also known as recall, measures the percentage of positive items that are correctly classified:

(45) $$Sensibility=\frac{tp}{tp+fn}.$$

Specificity measures the percentage of negative items that are correctly classified:

(46) $$Specificity=\frac{tn}{tn+fp}.$$

Precision refers the proximity of a prediction result to the actual value:

(47) $$Precision=\frac{tp}{tp+fp}.$$

F1-Score is the harmonic average of precision and recall:

(48) $$F1-Score=2\cdot \frac{Precision\cdot Recall}{Precision+Recall}\;.$$

Index Balanced Accuracy is a metric that is used to measure performance. It is given by the following:

(49) IBA=(1+α∗(Recall−Specificity))∗(Recall∗Specificity),

where α is the weight that regulates the *Dominance* of the class (commonly, a fixed value of 0.1). As such, the Dominance is obtained as follows:

(50) Dominance=Recall−Specificity.

### 3.3. Evaluation Using BUSI Ultrasound Dataset

When performing the genetic algorithm, we indicated that the following features were the most optimal to perform the classification.

As we can see in Table 3, the genetic and mutual information algorithms selected several deep type features, for example, Featuredeep23 and Featuredeep80. Also, they determined the HOG and LBP features, such as HOGLBPFaeture17. Additionally, the shape features, such as Eccentricity and Circularity, were relevant to classify benign- and malignant-type lesions.

The binary classification (malignant and benign) was performed (see Table 4) using the genetic and mutual information features selection algorithms, as well as by applying the IBA metric since, for the US dataset, there are only about half as many images of the malignant class in comparison with benign ones.

**Figure 14:**
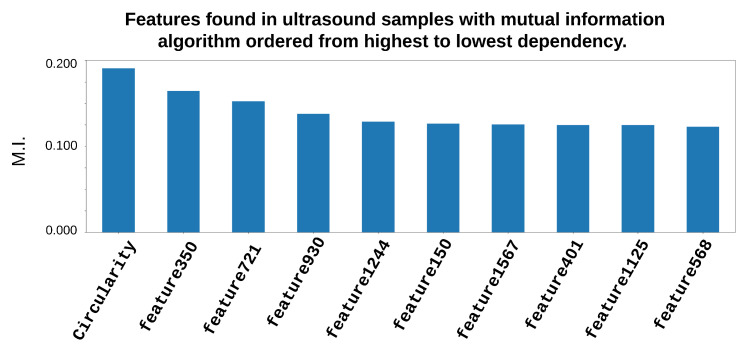
Features most representative from a US image, employing the genetic algorithm and mutual information algorithm fusion features.

Figure 14 shows the obtained features when the algorithm based on mutual information was used for feature selection from the US database.

One can see (Figure 14) that there were 10 features where the circularity was the most relevant or presented the most information to carry out the diagnosis, following deep type features such as Feature350, Feature721, etc. In this case, the features of HOG and LBP did not give relevant information to carry out the diagnosis in accordance with the selection of components as the most important features.

### 3.4. Evaluation Using Mammography Dataset Mini-DDSM

Next, let us present the results obtained for this dataset for binary labeling (malignant and benign). Genetic and mutual information selection algorithms obtained the features in Table 5 to perform the classification.

**Figure 15:**
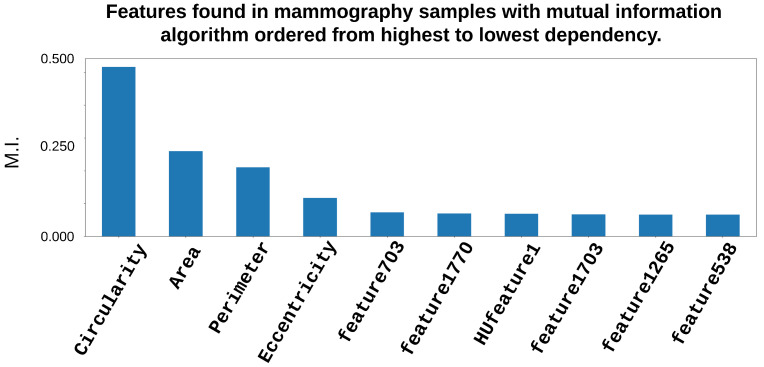
Most representative features from an MG image, determined by employing the genetic algorithm and mutual information.

In Figure 15, one can see the obtained features when the algorithm based on mutual information was employed in the selection of the most informative features from the MG database.

Observing Figure 15, one can see that the shape feature was the most relevant one, because the circularity was again in first place, followed by area, perimeter, and eccentricity. Additionally, among the deep type features, there were ones such as 721, 207, and, for HOG–LBP PCA-reduced features, there were HUfeature1 and HUfeature703.

Analyzing experimental results presented for this dataset in Table 6 and Figure 12, we can conclude that the proposed system, when the genetic and mutual information algorithms are used for feature selection procedure, followed by the XGBoost classifier, appears to demonstrate the best performance.

### 3.5. Significance Analysis Using Wilcoxon Test

The Wilcoxon test is a non-parametric statistical test performed by evaluating two groups. The observations within each group must be independent and identically distributed, and independence is assumed between the two groups [65,66,67,68]. The null hypothesis $H_{0}$ is taken as the starting point of the investigation and is not rejected unless the sample data seem to show that it is false. In such a case, there is a relationship between the parameters or the event investigated.

The null hypothesis means that there is no difference between the two groups of the population (in terms of central tendency), and, conversley, the research hypothesis tells us that there is a difference between the two groups of the population (regarding the central tendency).

The Wilcoxon test is defined as follows:

(51) $$W=\sum_{i=1}^{N}i\cdot V_{i},$$

where *W* is the sum of the *n* ranks of group 1; the ranks are determined based on the pooled sample of all *N* values.

To determine whether the difference between the population median and the hypothesized median is statistically significant, the calculated sum *W* is compared with the significance level. Usually, a significance level α of 0.05 (5%) works well. This significance level indicates a 5% risk of concluding that a difference exists when there is no actual difference. Thus, if the foundation level is less than α, the decision is to reject the null hypothesis (Reject $H_{0}$). In this case, we can conclude that the difference between the population median and the hypothesized median is statistically significant. However, if the *value*>α, the difference between the medians is not significantly different, meaning it is impossible to reject $H_{0}$.

We carried out this test since we had two groups, malignant and benign, where the features obtained were evaluated individually. For example, when we chose the feature of circularity for benign lesions, taking this as group one, and we took the feature of malignant circularity as group two, the results, according to the *p*-value obtained, were obtained, as one can see in Table 7.

We can conclude that most of the US and MG characteristics obtained by the employed selection methods (GA and MI) confirmed the rejection of the null hypothesis. Thus, these characteristics can serve in diagnostic analysis and decisions.

### 3.6. Evaluating Fusing the US and MG Images

In this section, we present (in Table 8) the results obtained when the features of the two databases were employed to be able to classify images from one or the other. The fusion of MG and US features was carried out, obtaining better results compared to each database analyzed individually, since there was more information about the lesions in general, having more data to analyze and more patterns to distinguish between one dataset and another, although, in both, circularity was one of the important features, as obtained from both the genetic algorithm and for mutual information algorithm.

**Figure 16:**
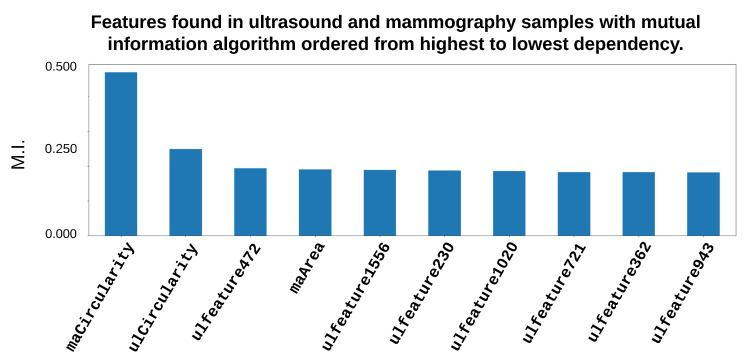
Most representative features from the fusion of US and MG images when employing the genetic and mutual information algorithms.

We used the results from performing the random undersampling procedure for 210 from each class, obtaining feature vectors of 420 × 2123 from the total features (Deep, HOG and LBP, Area, Perimeter, Eccentricity, Circularity), one for US and one for MG. Then, we concatenated the vectors, obtaining 420 × 4246 in a single dataset. The selection procedures for features were carried out by employing the genetic and mutual information algorithms. Then, 2 types of the feature vectors were again obtained, with 1 of 26 features selected by employing the mutual information algorithm and another one when the genetic algorithm for selection was used, resulting in 20 selected features. In Figure 16, one can observe the features that contributed the most important information when the genetic or mutual information algorithms were used during the selection stage in the proposed system. In this case, the designed system employed two databases: MG and US.

In the final evaluation stage, these fused feature vectors, with a size of 420×46, were divided for training—336 (80%) and for testing—84 (20%). Similar to the evaluation performed for separate US or MG datasets, the separation of the two classes (malignant and benign) was carried out via the three mentioned classifiers (shown in Table 9).

**Figure 17:**
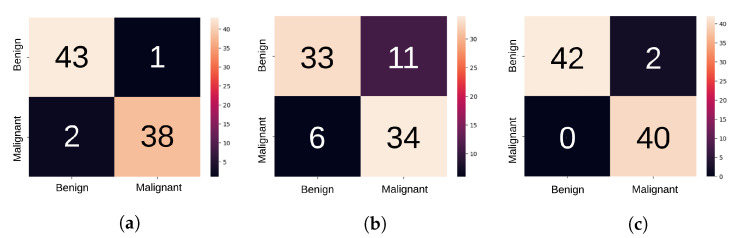
Confusion matrix when genetic and mutual information selection algorithms for features were employed for the compound (US + MG) dataset, (**a**) using XGBoost, (**b**) using MLP, and (**c**) using AdaBoost classifiers.

The confusion matrices obtained for each classifier are presented in Figure 17, where only malignant and benign lesions were classified, following the BI-RADS medical classification system. The normal category was discarded, similar to several authors [12,13] that used the same binary classification.

### 3.7. Comparison with State-of-the-Art Systems

In Table 10, we present the comparison results of the novel system with recent state-of-the-art systems.

In the literature, as we presented in the short review (Section 1), there are several promising techniques used in the diagnostics of lesions using MG images as well as US ones. In [11], the author used handcraft features such as HOG, LBP, and GLCM. However, the results obtained in [11] were deficient in comparison with those obtained by our proposed method because we extracted the different types of features and employed several classifiers. Daoud et al. in [13] used private US databases with 380 and 168 different types of features (texture and morphological), where some of them could have been redundant and did not give relevant information. Jabben et al. [14] proposed a system with the same BUSI US dataset. Their method demonstrated a better performance than our proposed method because they implemented a data augmentation strategy to balance the number of samples per class. In contrast, we employed the RUS technique for equilibrating the classes, confirmed via the IBA metric ACC measurement of 93%, justifying that our designed method is a balanced one that guarantees competitive performance without generating synthetic samples. In our opinion, a data augmentation strategy can duplicate data, presenting wrong criteria results. In [27], the authors proposed a BI-RADS-based system; however, they used a private database, which required labeling by the specialist. Their system employed EfficientNET, which is a more computationally intensive net in comparison with the DenseNET-201 architecture used in the proposed system. Heenaye et al. [15] used ResnNET-50; however, their system employed only deep learning features. Such an approach demands a large quantity of data to train this architecture from scratch. Oppositely, our system employed the transfer learning method and did not require a large quantity of data. The study in [17] used a customized deep neural architecture, where the main issue was the large data quantity needed to train the architecture from scratch. Because the datasets that they employed contain a reduced set of samples, they proposed to use data augmentation. Their system obtained an average accuracy of 93.2%, which is less than our proposed system. Additionally, their method was trained on a small quantity of data, so there is no guarantee that their architecture has the ability to extract the correct features. The system proposed in [18] employed the BUSI dataset, where the authors, because of the imbalance between the classes, performed data augmentation via generating synthetic samples that could duplicate data. Analyzing the system of Alsheikhy [19], we can see that their dataset was unbalanced—there were 1778 Malignant, 1408 Benign, and 185 Healthy images. Performing the classification process in such a dataset favored classification for the first two classes. For data fusion, our approach is competitive since it can be used for the MG database as well as for the US one. Another technique presented in [21] uses datasets with fewer images in comparison with those used for our proposed system. The databases INBreast and mini-MIAS present fewer images, and, even after augmentation, there are not sufficient data to guarantee the necessary volume of images. Their method used only deep learning features and did not employ handcraft features that could provide relevant information, as proposed in our designed system. Finally, systems presented in [12,20] employed the MIAS Breast dataset and used handcraft and deep learning strategies to classify MG images. The handcraft approach [12] achieved a 92.16% accuracy using frequency-based features, depending on the correct preprocessing of the ROI image. Moreover, the deep learning strategy [20] employed several CNNs and, finally, a Graph Convolutional Network (GCN) to unify and select the best features for the classification. However, this approach required high-performance hardware.

In this study, during experimental tests, two datasets [22,24], commonly used by several authors in the state-of-the-art [12,13,15,17,20], were evaluated. Table 10 shows the performance comparison of the designed systems versus different proposals published in the literature, which also used binary classification [11,12,13,15,16,17,20,21]. Different systems used the same datasets or subsets thereof [12,17,20], demonstrating outstanding performance [11,16,19], although several proposals did not provide access to their private datasets.

As one can see in Table 10, the proposed system surpasses the evaluation metrics of most existing systems by employing a lightweight strategy. The main goal of the DBFS_GMI CAD system is to be applicable to two different types of medical imaging studies. According to our knowledge, there is no other system that uses a compound dataset (both MG and US images), and, as can be seen in this study, this novel approach can increase the quality of the classification. Furthermore, the designed system uses a novel strategy for fusing different kinds of features to guarantee the classification of the lesions, providing a second opinion to a specialist.

## 4. Discussion

As one can see, the circularity feature is selected by both the genetic algorithm and by the mutual information selection procedure as the most informative for performance. This feature is defined in BI-RADS for mass classification as the principal in order to select if a lesion is benign or malignant, since a more uneven shape, i.e., a lower circularity, indicates a higher risk of malignancy. On the contrary, if it has a regular shape, it is more circular, indicating a lower risk of malignancy. In addition, the designed system relies on other shape features, such as eccentricity or area, that are important for classification. The feature selection and fusing algorithms also select those of the deep type features and select the HOG and LBP features. These features individually serve as a support in the classification but disappear in the merger since they are not the most relevant within the data merger. Finally, the novel system has been evaluated employing the metrics that support our results, such as IBA, and using tools such as random undersampling, providing justificiation that the novel system appears to demonstrate the best performance in comparison with state-of-the-art techniques. In contrast to the designed framework, analyses of different systems do not consider metrics such as IBA. Additionally, the designed system uses information according to standards based on medical information, where the commonly applied features are obtained, such as shape and texture. Most of the systems only obtain handcraft features. However, some of these systems are developed without defining the ROI of a lesion. As well, some authors use only deep type features, while our novel system generalizes the features through fusion from two datasets, where competitive results are obtained compared with systems that only use the images from one dataset.

## 5. Conclusions

This study has developed a new system to diagnose breast cancer, where two medical examinations, MG and US images, are employed. The designed hybrid system uses the CNN architecture for extracting deep learning features and traditional methods which perform several handcraft features that follow from the medical properties of disease, with the purpose of being later fused via statistical criteria. During the following stages, the proposed technique concatenates the features of both studies, forming the lis tof features that can classify any of the two datasets and diagnose a lesion as either benign or malignant. During the fusion stage, where deep learning and handcraft features are analyzed, the genetic and mutual information selection algorithms are employed; next, several classifiers (XGBoost, AdaBoost, MLP) are applied, choosing the best information group from among the features. The performance results of the designed system are satisfactory, since the percentages obtained for commonly used quality metrics are competitive with those from state-of-the-art systems. The novel system can help specialist doctors in obtaining a second opinion on their diagnosis. In future investigations, we plan to employ the designed fusion approach to other medical images, for example, MRI, CT, or SPECT.

## Figures and Tables

**Table 1:** Distribution of images in *mini_DDSM* and *BUSI* databases.

	TRAIN		TEST	
**Dataset**	**Benign**	**Malignant**	**Benign**	**Malignant**
BUSI	349	168	88	42
mini_DDSM	387	317	98	79

**Table 2:** Evaluation of the *genes* through 35 iterations.

Gen	$N_{\mathrm{vals}}$	Accuracy	Gen	$N_{\mathrm{vals}}$	Accuracy
0	500	0.9023	10	304	0.9273
1	310	0.9057	15	301	0.9273
2	282	0.9068	20	297	0.9307
3	285	0.9170	25	320	0.9307
4	297	0.9170	30	299	0.9352
5	301	0.9204	35	291	0.9352

**Table 3:** US features extracted and selected by employing the genetic algorithm and mutual information algorithm fusion features.

Featuredeep23	Featuredeep774	Featuredeep1495	HOGLBPFEATURE106
Featuredeep80	Featuredeep805	Featuredeep1595	HOGLBPFEATURE147
Featuredeep485	Featuredeep920	Featuredeep1716	Eccentricity
Featuredeep508	Featuredeep1045	Featuredeep1810	Circularity
Featuredeep665	Featuredeep1301	HOGLBPFEATURE17	

**Table 4:** Performance results for criteria when genetic and mutual information selection algorithms for features were employed for US images.

Classifier	Accuracy	Precision	Recall	Specificity	F1-Score	IBA
XGBoost	96.1%	96.0%	96.0%	96.0%	96.0%	92.0%
MLP	90.0%	91.0%	90.0%	87.0%	90.0%	77.0%
AdaBoost	93.8%	94.0%	94.0%	92.0%	94.0%	87.0%

**Table 5:** MG features extracted and selected by employing the genetic algorithm and mutual information algorithm fusion features.

Featuredeep72	Featuredeep257	Featuredeep1199	Featuredeep1561
Featuredeep1818	HUfeature100	Featuredeep207	Featuredeep490
Featuredeep1305	Featuredeep1572	Featuredeep1895	Area
Featuredeep225	Featuredeep508	Featuredeep1397	Featuredeep1695
Featuredeep1908	Perimeter	Featuredeep244	Featuredeep863
Featuredeep1448	Featuredeep1772	HUfeature37	Circularity
Featuredeep252	Featuredeep887	Featuredeep1526	Featuredeep1774
HUfeature48			

**Table 6:** Performance results for criteria when genetic and mutual information selection algorithms for features are employed for MG images.

Classifier	Accuracy	Precision	Recall	Specificity	F1-Score	IBA
XGBoost	93.8%	94.0%	94.0%	93.0%	94.0%	88.0%
MLP	90.0%	91.0%	90.0%	87.0%	90.0%	77.0%
AdaBoost	86.1%	86.0%	86.0%	85.0%	86.0%	73.0%

**Table 7:** Obtained *p*-values for selected features and evaluated for the groups Benign and Malignant, via Wilcoxon test.

MG Features			US Features		
Eccentricity	$p=4.222\times 10^{-26}$	Different distribution (Reject $H_{0}$)	Circularity	$p=4.222\times 10^{-26}$	Different distribution (Reject $H_{0}$)
Area	$p=7.988\times 10^{-36}$	Different distribution (Reject $H_{0}$)	Eccentricity	$p=2.896\times 10^{-5}$	Different distribution (Reject $H_{0}$)
Circularity	$p=3.019\times 10^{-53}$	Different distribution (Reject $H_{0}$)	HUFeature147	p=0.456	Same distribution (Fail to reject $H_{0}$)
HUfeature1	$p=1.430\times 10^{-17}$	Different distribution (Reject $H_{0}$)	Feature230	$p=2.169\times 10^{-19}$	Different distribution (Reject $H_{0}$)
Feature702	p=0.034	Different distribution (Reject $H_{0}$)	Feature362	$p=2.308\times 10^{-19}$	Different distribution (Reject $H_{0}$)
HUfeature37	p=0.00042	Different distribution (Reject $H_{0}$)	Feature721	$p=6.553\times 10^{-20}$	Different distribution (Reject $H_{0}$)

**Table 8:** MG and US features extracted and selected by employing genetic algorithm and mutual information algorithm fusion features.

maCircularity	ulCircularity	ulfeature472	maArea	ulfeature1556	HUfeature100
ulfeature230	ulfeature1020	ulfeature721	ulfeature362	ulfeature943	Area
ulfeature388	ulfeature930	ulfeature432	ulfeature89	ulfeature1141	Perimeter
ulfeature578	ulfeature838	ulfeature341	ulfeature778	ulfeature837	Circularity
ulfeature475	ulfeature350	ulfeature810	ulfeature265	ulfeature608	ulfeature969

**Table 9:** Performance criteria results when genetic and mutual information selection algorithms for features were employed for the compound (US + MG) dataset.

Classifier	Accuracy	Precision	Recall	Specificity	F1-Score	IBA
XGBoost	96.4%	96.0%	96.0%	96.0%	96.0%	93.0%
MLP	79.7%	80.0%	80.0%	80.0%	80.0%	64.0%
AdaBoost	97.6%	98.0%	98.0%	98.0%	98.0%	95.0%

**Table 10:** Performance comparison with state-of-the-art systems.

Proposed System	DATASET	ACCURACY	PRECISION	SENSIBILITY	SPECIFICITY	F1-Score	IBA
Wei et al. [11]	Privative	86.67%	-	92.45%	78.38%	-	-
Zhang et al. [12]	MIAS	92.16%	-	92.22%	92.10%	-	-
Daoud et al. [13]	BUSI	96.1%	-	95.7%	96.3%	-	-
Jabeen et al. [14]	BUSI	99.1%	99.2%	99.2%	-	99.2%	-
Heenaye et al. [15]	CBIS–DDSM	88.0%	-	-	-	-	-
Tsai et al. [16]	Privative	94.22%	-	95.31%	99.15%	-	-
Muduli et al. [17]	MIAS	96.55%	-	97.28%	95.92%	-	-
	DDSM	90.68%	-	92.72%	88.21%	-	-
	INbreast	91.28%	-	99.43%	83.13%	-	-
	BUS-1	100%	-	100.00%	100.00%	-	-
	BUS-2	89.73%	-	93.33%	86.14%	-	
Raza et al. [18]	BUSI	99.35%	99.6%	99.66%	-	99.5%	-
Alsheikhy et al. [19]	Privative	99.14%	99.68%	99.4%	94.87%	99.54%	-
Zhang et al. [20]	MIAS	96.1%	-	96.2%	96%	-	-
Nagwan et al. [21]	DDSM	98.6%	-	99.62%	-	-	-
Proposed DBFS_GMI	DDSM	92.0%	93.0%	92.0%	92.0%	93.0%	84.0%
Proposed DBFS_GMI	BUSI	96.0%	96.0%	96.0%	96.0%	96.0%	92%
Proposed DBFS_GMI	Composed by BUSI and DDSM	*97.0%*	*98.0%*	*98.0%*	*98.0%*	*98.0%*	*95.0%*

## Data Availability

The data and code presented in this study are available on request to the corresponding author for academic purposes.

## Abbreviations

The following abbreviations are used in this manuscript:

US	Ultrasound Image
MG	Mammography Image
ROI	Region of Interest
DL	Deep Learning
CAD	Computer-Aided Diagnostic
SVM	Support Vector Machine
CNN	Convolutional Neural Network
TF	Transfer Learning
MI	Mutual Information
